# A Quantitative Analysis of the Distribution of CRH Neurons in Whole Mouse Brain

**DOI:** 10.3389/fnana.2017.00063

**Published:** 2017-07-25

**Authors:** Jie Peng, Ben Long, Jing Yuan, Xue Peng, Hong Ni, Xiangning Li, Hui Gong, Qingming Luo, Anan Li

**Affiliations:** ^1^Collaborative Innovation Center for Biomedical Engineering, Wuhan National Laboratory for Optoelectronics, Huazhong University of Science and Technology Wuhan, China; ^2^Britton Chance Center, School of Engineering Sciences, Huazhong University of Science and Technology Wuhan, China; ^3^MOE Key Laboratory for Biomedical Photonics, School of Engineering Sciences, Huazhong University of Science and Technology Wuhan, China

**Keywords:** corticotropin-releasing hormone, neuron, three-dimensional reconstruction, automatic segmentation, image processing, brain-wide dataset, optical imaging

## Abstract

Corticotropin-releasing hormone (CRH), with widespread expression in the brain, plays a key role in modulating a series of behaviors, including anxiety, arousal, motor function, learning and memory. Previous studies have focused on some brain regions with densely distributed CRH neurons such as paraventricular hypothalamic nucleus (PVH) and bed nuclei of the stria terminalis (BST) and revealed some basic structural and functional knowledge of CRH neurons. However, there is no systematic analysis of brain-wide distribution of CRH neurons. Here, we performed a comprehensive study of CRH neurons in *CRH-IRES-Cre;Ai3* mice via automatic imaging and stereoscopic cell counting in a whole mouse brain. We acquired four datasets of the CRH distributions with co-localized cytoarchitecture at a voxel resolution of 0.32 μm × 0.32 μm × 2 μm using brain-wide positioning system (BPS). Next, we precisely located and counted the EYFP-labeled neurons in different regions according to propidium iodide counterstained anatomical reference using Neuronal Global Position System. In particular, dense EYFP expression was found in piriform area, BST, central amygdalar nucleus, PVH, Barrington’s nucleus, and inferior olivary complex. Considerable CRH neurons were also found in main olfactory bulb, medial preoptic nucleus, pontine gray, tegmental reticular nucleus, external cuneate nucleus, and midline thalamus. We reconstructed and compared the soma morphology of CRH neurons in 11 brain regions. The results demonstrated that CRH neurons had regional diversities of both cell distribution and soma morphology. This anatomical knowledge enhances the current understanding of the functions of CRH neurons. These results also demonstrated the ability of our platform to accurately orient, reconstruct and count neuronal somas in three-dimension for type-specific neurons in the whole brain, making it feasible to answer the fundamental neuroscience question of exact numbers of various neurons in the whole brain.

## Introduction

Corticotropin-releasing hormone (CRH) is an important broadly expressed neuropeptide which has neuroendocrine and neurotransmitter properties (Vale et al., 1981; Young, 1992). CRH plays a key role in the regulation of the hypothalamic–pituitary–adrenal (HPA) axis through stimulating the synthesis and secretion of adrenocorticotropin. CRH-expression neurons also participate in various functional activities in discrete brain regions. Therefore, identifying the brain-wide distribution of CRH can increase the current understanding of the functions of CRH in various neural circuits and activities.

Genetic techniques provide access for targeting specific neurons according to the molecular expression of CRH in the brain. Using these labeling methods, the distribution features of CRH in the rodent brain have been extensively explored in some brain regions and nuclei, such as the amygdala (De Francesco et al., 2015), olfactory bulb (Huang et al., 2013; Garcia et al., 2016) and bed nuclei of the stria terminalis (BST) (Nguyen et al., 2016). Furthermore, the expression patterns of CRH in whole mouse brains had been qualitatively described (Alon et al., 2009; Kono et al., 2016). These studies acquired data from images of histological sections and analyzed these images through two-dimensional cell counting. Manual sectioning and imaging is time-consuming and laborious. Counting cells in interval-sampling planar images can be inaccurate due to a lack of data continuity and possible errors in counting axial-overlapping cells. Therefore, the comprehensive organization of brain-wide CRH network has not been systematically and quantitatively analyzed.

Fortunately, advances in whole-brain optical imaging and stereological cell counting have made it feasible to quantify the cell distribution and number of CRH neurons in the whole brain. Light-sheet illumination microscopy, serial two-photon tomography (Ragan et al., 2012), and fluorescence micro-optical sectioning tomography (fMOST) serial technologies (Gong et al., 2013, 2016; Zheng et al., 2013) all have demonstrated capabilities of acquiring brain-wide datasets at different resolutions. Particularly, brain-wide positioning system (BPS) (Gong et al., 2016), the latest model of fMOST and a dual-color precision imaging system, enabled the acquisition of whole morphology and co-located landmarks of labeled neurons in the whole brain at sub-cellular resolution. Different algorithms (Long et al., 2009; Al-Kofahi et al., 2010; Quan et al., 2013) have been developed to detect, locate and count neuronal somas in three-dimension. Among these methods, Neuronal Global Position System (NeuroGPS) (Quan et al., 2013), a three-dimensional automatic cell locating tool, has the advantages of eliminating interference from the thick dendritic trunks and robust performance in recognizing neurons of diverse sizes/shapes and intensity.

Here, we used BPS and NeuroGPS to compose a whole-brain cell counting platform and mapped the distribution of CRH expression in *CRH-IRES-Cre;Ai3* EYFP mouse brains. We acquired four brain-wide datasets of EYFP-labeled CRH neurons with co-located propidium iodide (PI, a nuclear dye) stained cytoarchitecture at the voxel resolution of 0.32 μm × 0.32 μm × 2 μm using BPS. We detected and counted CRH neurons automatically. Subsequently, we segmented distinct regions from PI-stained signals according to a standard reference brain atlas, and quantified the distribution of CRH neurons in each region. The quantitative results showed that CRH neurons were widely distributed with different density in the brain regions. Densities of CRH neurons in paraventricular hypothalamic nucleus (PVH), Barrington’s nucleus (B), and inferior olivary complex (IO) were significantly higher than that in other areas. Relative dense CRH neurons were found in piriform area (PIR), BST, central amygdalar nucleus (CEA) locally or in the whole nucleus. These results were consistent with previous study (Alon et al., 2009; Kono et al., 2016). Additionally, considerable CRH neurons were found in the brain regions with few attention in previous study, including main olfactory bulb (MOB), medial preoptic nucleus (MPN), pontine gray (PG), tegmental reticular nucleus (TRN), external cuneate nucleus (ECU), and midline thalamus. We also reconstructed and quantitatively analyzed the soma shape of CRH neurons in specific regions. The brain-wide distribution and soma morphological features of CRH neurons potentially facilitates the current understanding of CRH neurons in brain functions. Furthermore, these results also demonstrated the ability of this platform to precisely image and orient neurons, reconstruct soma morphology and accurately count somas for given-type neurons in the whole brain. The demonstration of CRH expression illustrated that this platform could potentially become a routine tool for neuroscience study.

## Materials and Methods

### Animals

CRH-Cre transgenic mice (Taniguchi et al., 2011) and EYFP Cre reporter Ai3 mice (Madisen et al., 2009) were obtained from the Josh Huang Lab (CSHL, Cold Spring Harbor, NY, United States) and Jackson Laboratories (JAX Mice stock number: 007903), respectively. *CRH-IRES-Cre;Ai3* mice were generated by crossing CRH-Cre male mice with EYFP Cre reporter Ai3 female mice. Four 3-month-old *CRH-IRES-Cre;Ai3* male mice were used for cell counting, and a 2-month-old *CRH-IRES-Cre;Ai3* male mouse was used for immunostaining. The mice were housed on a 12-hour light/dark cycle with food and water ad libitum. All the animal experiments were performed according to the procedures approved by the Institutional Animal Ethics Committee of Huazhong University of Science and Technology.

### Tissue Preparation

All histological procedures have been previously described (Yang et al., 2013; Gong et al., 2016). Briefly, the mice were anesthetized with a 1% solution of sodium pentobarbital and subsequently intracardially perfused with 0.01 M PBS (Sigma–Aldrich, United States), followed by 4% paraformaldehyde (PFA, Sigma–Aldrich, United States) and 25% sucrose in 0.01 M PBS. Subsequently, the brains were excised and post-fixed in 4% PFA at 4°C for 24 h. After fixation, each intact brain was rinsed overnight at 4°C in 0.01 M PBS and stored until use.

For whole brain imaging, the brains were dehydrated in a graded ethanol series (50, 70, and 95% ethanol at 4°C for 1 h each). After dehydration, the brains were immersed in a graded glycol methacrylate (GMA, Ted Pella, USA) series, including 0.2% SBB (70, 85, and 100% GMA for 2 h each and 100% GMA overnight at 4°C). Subsequently, the samples were impregnated in a prepolymerization GMA solution for 3 days at 4°C and embedded in a vacuum oven at 48°C for 24 h. The 100% GMA solution comprised 67 g of A solution, 2.8 g of deionized water, 29.4 g of B solution, 0.2 g of SBB, and 0.6 g of AIBN as an initiator. The 70 and 85% GMA solutions (wt/wt) were prepared from 95% ethanol and 100% GMA.

For immunostaining, the brains were sectioned at 50 μm on a vibratome (Leica VT 1200S, Germany) and all sections were stored at 4°C.

### Immunohistochemistry

A series of sections were selected from the olfactory bulb to the brainstem of the whole mouse brain for immunohistochemistry. The free-floating sections were permeabilized using 0.3% Triton-X 100 in PBS and blocked with 5% Bovine Serum Albumin (BSA, Sigma–Aldrich, United States) for 1 h at 37°C. Subsequently the sections were incubated with rabbit anti-CRH primary antibody (Bachem, T4037, at 1:1000 dilution) overnight at 4°C. After washing three times with PBS for 10 min, the sections were incubated with Alexa Fluor 594 goat anti-rabbit IgG secondary antibody (Invitrogen, A-11037, at 1:1000 dilution) for 2 h at 37°C. After washing steps, the sections were mounted and cover slipped with Fluoro-gel. The representative images were acquired using a confocal microscope (LSM 710, Zeiss, Jena, Germany).

### Whole-Brain Imaging

The resin-embedded brain samples were imaged using a homemade BPS system (Gong et al., 2016). Briefly, BPS was a three-dimensional dual-color epifluorescence microscope and involved a brain-wide tomography via structured illumination. During the imaging, the sample was immersed in water and a water immersion objective (1.0 NA, 20X) was used. Both PI signals and specific labeled fluorescence signals were simultaneously detected via two scientific complementary metal-oxide semiconductor (sCMOS) cameras, a new kind of scientific camera with highly sensitive and high-speed. A precise 3D translational stage was employed to move the sample to perform scanning. We acquired an image of the top surface of the sample using the microscope and subsequently sectioned the sample to remove the imaged tissue at 2 μm. The imaging-sectioning cycle was maintained layer by layer until the data acquisition was completed. Subsequently we obtained a continuous image stack of the whole brain. The mechanical translation stages accurately controlled the movement of the sample and ensured the auto-alignment of the dataset. EYFP-labeled CRH neurons and PI-stained cytoarchitecture datasets of each *CRH-IRES-Cre;Ai3* mouse brain were simultaneously acquired at a voxel resolution of 0.32 μm × 0.32 μm × 2 μm in 3 days. The coronal slice number and original data size of one channel dataset were approximately 5200 and 4 TB, respectively.

### Data Processing and Analysis

We segmented 140 brain regions in the *CRH-IRES-Cre;Ai3* mouse according to Allen CCFv3 (Kuan et al., 2015). The dataset was rotated and resampled at a voxel resolution of 10 μm × 10 μm × 10 μm for registering to the template dataset of Allen CCFv3. We performed an affine transformation and a symmetric image normalization (Rueckert et al., 1999; Klein et al., 2009; Ni et al., 2015) in Advanced Normalization Tools (ANTS) (Klein et al., 2009; Avants et al., 2011) to achieve the three-dimensional co-registration of the PI-stained dataset with the Allen template dataset. Subsequently, we segmented the brain regions in the PI-stained dataset by extracting the contours of brain regions in the Allen template dataset. Three skilled persons performed back-to-back manual validation and amendment in Amira software (v 6.1.1, FEI, France). All the segmentation results were saved as 3D-TIFF files.

We located and counted EYFP-labeled CRH neurons in the whole brain using NeuroGPS software. Since the entire dataset was oversized for the memory capability of a graphics workstation, we divided the dataset into approximately 50,000 blocks of 512 × 512 × 512 voxels with 50-voxel guard zones. Subsequently, we performed the automatic cell counting on each block. The centers of the neurons were automatically recognized, and the contours of the somas were segmented. The morphological features of the somas, including the longest, shortest, and average radii, the surface area and the cell volume, were measured, and the corresponding longest to shortest radii ratio was calculated. We merged the results from all the blocks as the whole-brain results.

To evaluate the accuracy of automatic locating and counting, we compared the results of automatic recognition with manual positioning. Back-to-back manual validation done in Amira by three skilled persons was considered as the ground truth. Manual counting was done using stereological visualization and cell counting in individual serial sections in evaluated data blocks. In each section, all cell bodies were manually marked and subsequently checked in the adjacent sections to avoid missed or repeated identification. We then visualized the data blocks and located marked cells in three-dimension to further check manual counting results. The above process was done iteratively until all the cells were checked and located precisely in stereological visualization. Also, to avoid possible subjective deviations or errors, all the evaluated data blocks were identified and checked by three persons. We calculated the two parameters of recall and precision (He et al., 2014) to evaluate the location accuracy. B1 and B2 represented the total number of the manually and automatically recognized neurons, respectively. B represented the number of correctly detected neurons by NeuroGPS after compared to the ground truth. The recall *R* was defined as *R* = B/B1, while the precision P was defined as *P* = B/B2. We randomly selected twelve evaluated data cubes of 300 μm × 300 μm × 300 μm from a whole-brain dataset, and calculated *R* and *P* for each data cube.

We quantified the CRH neuron distribution in 140 discrete brain regions across the entire extent of the whole brain. The neuron number in each region was counted according to their locations. We defined the neuron density of a specific brain region as the neuron number divided by the region volume. For each region, we added all the voxels in the segmented region contour and obtained the region volume after multiplying total voxel number by the voxel resolution. We further quantified the distribution feature using nuclei distances in the specific regions. We defined the nuclei distance in one brain region as the mean value of all the distances of the neurons in the region. The distance of one neuron was defined as distance from the position of the neuron to the nearest position of the other neuron in the same brain region.

We analyzed the morphological similarities and differences of CRH neuron somas in 11 discrete brain regions. We randomly selected a data cube of 200 μm × 200 μm × 200 μm in each region. To determine the main features of the CRH soma shape, we conducted a principal component analysis (PCA) (Jolliffe, 2002) of the soma morphological features. Since there were only 2 neurons in the data cube in the cerebellum (CB), we removed this data cube from the PCA analysis. PCA revealed the first two components by eigenvalues which explained 66.7 and 27.9% of the total variance, respectively. After orthogonal transformation by PCA and min-max normalization, a new feature space, including these two main features was obtained. We selected the average point as the original point and determined 80% of the closest points according the Euclidean distance (Danielsson, 1980) to eliminate deviation points. We calculated the intra-group standard deviation (SD) of the two main features of the concentrated points to estimate the similarities and differences of the soma morphology in each region. Lower SD indicated higher similarity of soma morphology in the regions. Moreover, we assessed the statistical significance of soma morphology between different regions using analysis of variance (ANOVA). *P* values <0.05 were considered significant.

## Results

### Immunohistochemistry for EYFP Expression Validation

**FIGURE 1:**
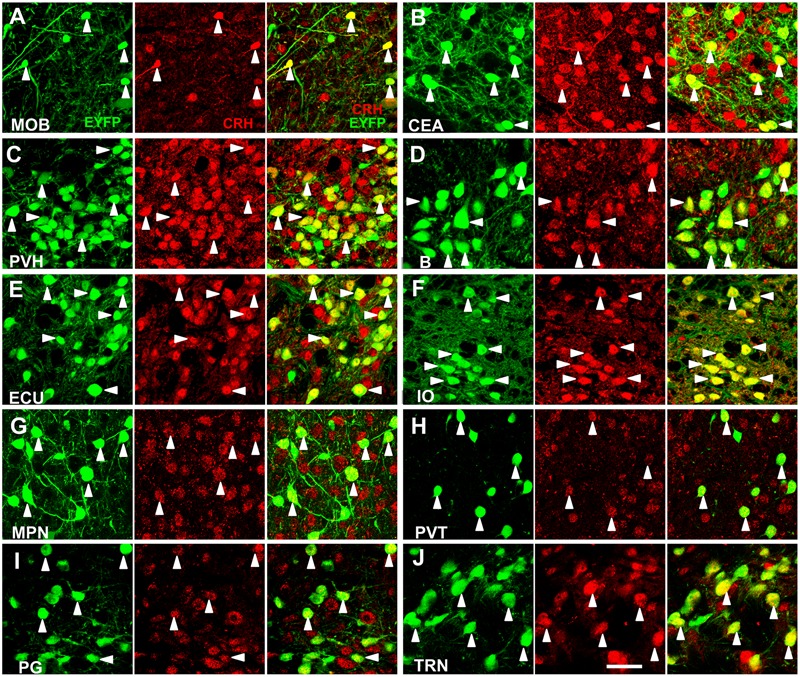
Specific EYFP-expression of *CRH-IRES-Cre;Ai3* mouse brain. **(A–J)** Representative photomicrographs depicting CRH neurons expressing EYFP (green), CRH immunostaining (red) and their merged images in MOB, CEA, PVH, B, ECU, IO, MPN, PVT, PG, and TRN, respectively, in a *CRH-IRES-Cre;Ai3* mouse brain. The arrowheads show EYFP and CRH double-positive neurons. Scale bar: 40 μm. Abbreviations: main olfactory bulb (MOB), central amygdalar nucleus (CEA), paraventricular hypothalamic nucleus (PVH), Barrington’s nucleus (B), external cuneate nucleus (ECU), inferior olivary complex (IO), medial preoptic nucleus (MPN), paraventricular nucleus of the thalamus (PVT), pontine gray (PG), and tegmental reticular nucleus (TRN).

We systematically analyzed the co-localization of EYFP expression with CRH immunoreactivity in the brain regions which had considerable neurons to evaluate the accuracy of targeting CRH neurons. We selected ten brain regions with relatively dense distributed neurons over the whole brain to validate EYFP expression (**Figure 1**). The majority (∼90%) of fluorescence-labeled neurons were stained positive in these areas. Conversely, the majority (∼80%) of CRH stained neurons were YFP positive in the MOB, PVH, B, ECU, IO, paraventricular nucleus of the thalamus (PVT), PG, and TRN, while relatively lower (∼50%) co-labeling was observed in CEA and MPN. These results demonstrated the relative accuracy of the CRH driver mouse line to map the distribution of CRH neurons in *CRH-IRES-Cre;Ai3* mouse brain.

### Mapping the Brain-Wide CRH Distribution

**FIGURE 2:**
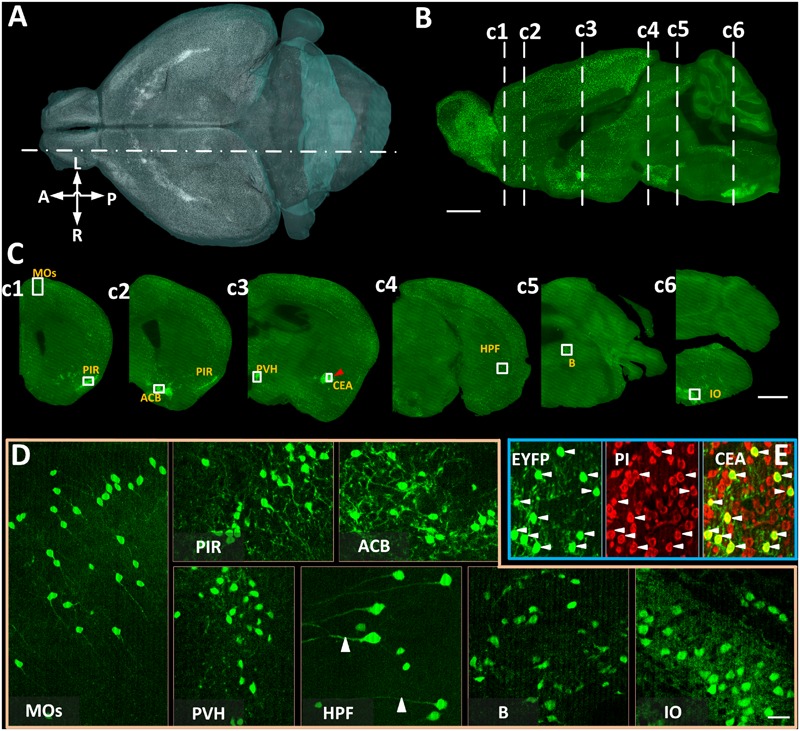
The brain-wide distribution of CRH neurons imaged by BPS. **(A)** Three-dimensional rendering of EYFP-labeled CRH neurons. Resampling at 2 μm × 2 μm × 2 μm. **(B)** Sagittal reconstruction of the maximum intensity projection at the location indicated by a dot dash line in **(A)** in the *CRH-IRES-Cre;Ai3* mouse brain. Projection thickness was 100 μm. **(C)** The 50-μm-thick maximum intensity projections of the coronal slices indicated by dash lines in **(B)**, respectively. **(D)** Enlarged views of EYFP-labeled CRH neurons of the area indicated with text annotations and white boxes in **(C).** Images of MOs and HPF were the maximum intensity projections of 50 μm, reflecting the sparse distribution of CRH neurons. Images of PIR, ACB, PVH, B, and IO were original data of a single slice. **(E)** Original images of EYFP-labeled CRH neurons, PI-stained cytoarchitecture and the merge of enlarged views of the area indicated by a red arrowhead and a white box in CEA in **(c3).** Scale bars: **(A,B)** 1 mm, **(C)** 1 mm, and **(D)** 30 μm. Secondary motor area (MOs), piriform area (PIR), nucleus accumbens (ACB), PVH, CEA, hippocampal formation (HPF), B, and IO.

We acquired coronal images of CRH neurons with co-located cytoarchitecture in 4 *CRH-IRES-Cre;Ai3* mouse brains using BPS (**Figure 2**). Benefiting from data continuity, we reconstructed a volume rendering (**Figure 2A**) and a sagittal section (**Figure 2B**) of the typical whole-brain dataset. Both results showed that EYFP-labeled CRH neurons are widely spread throughout the whole brain with a diversity of cell density in various brain areas. We observed that EYFP-labeled CRH neurons were densely distributed in the PIR, nucleus accumbens, PVH, CEA, B, and IO, and relatively sparsely distributed in secondary motor area and hippocampal formation (**Figure 2C**), consistent with previous studies (Alon et al., 2009; Kono et al., 2016). A voxel resolution of 0.32 μm × 0.32 μm × 2 μm facilitated the distinction of not only individual EYFP-labeled CRH neurons but also EYFP-labeled neural fibers in both sparsely and densely distributed brain areas (**Figure 2D**). Real-time counterstaining provided a cytoarchitecture for subsequent brain region recognition in the data analysis. The co-localization of EYFP-labeled CRH somas and PI-stained nuclei is shown in **Figure 2E**, demonstrating the orientation accuracy of cellular resolution. These results illustrated that BPS could be used to image the whole brains and provide high-resolution datasets for subsequent automatic cell counting.

### Locating CRH Neurons in the Whole Brain

**FIGURE 3:**
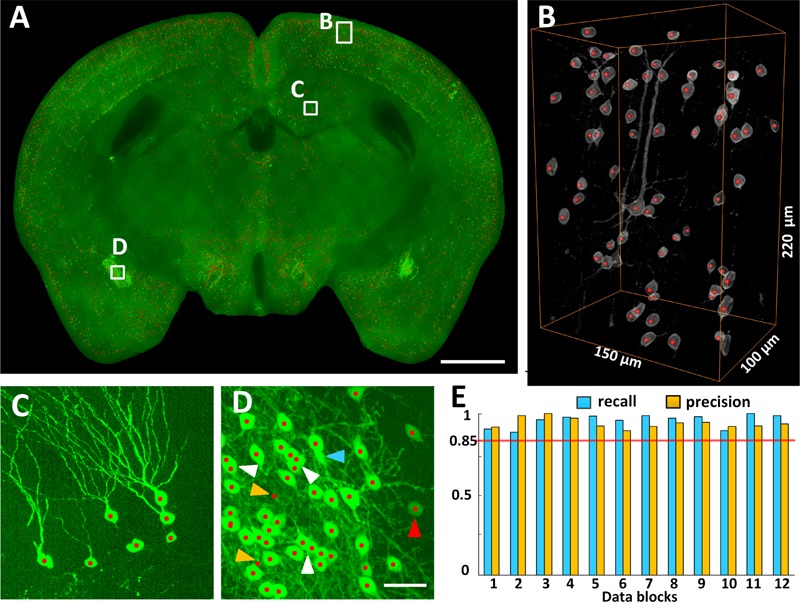
Locating EYFP-labeled CRH neurons in the whole *CRH-IRES-Cre;Ai3* mouse brain. **(A)** Locating neurons in 100 μm typical image stacks, presented in maximum intensity projection. **(B)** Three-dimensional reconstruction and **(C,D)** projection views of the data cubes indicated as white boxes in **(A)**, respectively. Gray and green signals represent CRH neuronal somas. Red dots represent the recognized centers of CRH neurons. The white, red, blue and orange arrowheads indicated the axial-overlapping neurons, low intensity neurons, error recognition neurons and misidentification neurons, respectively. **(E)** Counting accuracy. Blue and orange represent the recall and precision (*n* = 12), respectively. Each data cube was randomly selected at a size of 300 μm × 300 μm × 300 μm. Scale bars: **(A)** 1 mm and **(C,D)** 50 μm.

The locations of CRH neurons in the whole mouse brain were automatically recognized using NeuroGPS (**Figure 3**). **Figure 3A** shows a 100-μm projection of the overlap of CRH neurons (Green) and their soma centers (red) at the hippocampal formation coronal plane. The three-dimensional visualization of stereoscopic cell counting in **Figure 3B** shows a recall and precision of nearly 100% using automatic recognition. **Figure 3C** is an enlarged view of **Figure 3A** (indicated by a square box and text annotation). The results demonstrated that we could eliminate the interference from thick dendritic trunks (**Figure 3C**). Even for densely distributed CRH neurons, the recall and precision were still as high as 98 and 96%, respectively (**Figure 3D**). The white arrowheads in **Figure 3D** indicate that some overlapped cells in the projection view were recognized using stereoscopic cell counting (red dots). The finding demonstrated that counting cells in the three-dimensional dataset was more accurate than in the projection images. Some neurons would be inaccurately located due to misidentification of touching cells and error recognition in areas with strong background. Furthermore, we randomly selected 12 data cubes of 300 μm × 300 μm × 300 μm from the whole brain and evaluated their recall and precision (**Figure 3E**). The highest, lowest and average recall values were 100, 88, and 95%, respectively, and the highest, lowest and average precision values were 100, 89, and 93%, respectively. These results demonstrated that accurate locating provided cell counts for the following distribution analysis.

### Segmenting Brain Regions for Anatomical Orientation

**FIGURE 4:**
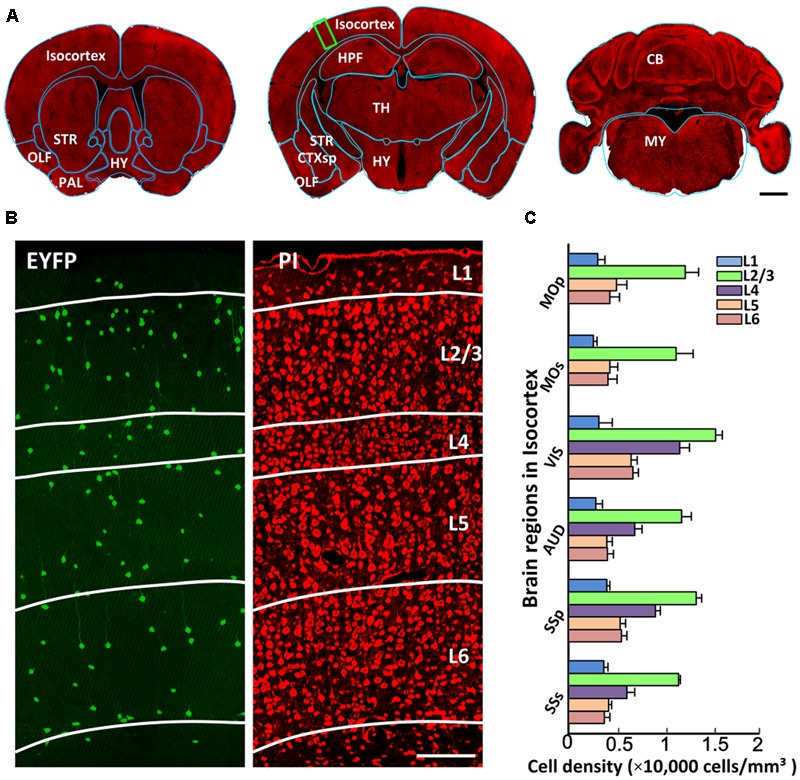
The segmentation of anatomical regions. **(A)** A 10-μm maximum intensity projection of representative PI-stained coronal slices in the same mouse brain. The cyan lines represent the segmented results using the ANTS tool. **(B)** EYFP-labeled CRH neurons and PI-stained cytoarchitecture of the enlarged view of the area indicated by a green rectangular box in **(A)**. Green represents the 100 μm maximum intensity projection of EYFP-labeled CRH neurons. Red represents the 2 μm original data of PI-stained cytoarchitecture. White lines represent the laminated contours of the cortex. **(C)** Quantitative statistics of EYFP-labeled CRH neurons in different layers in MOp, MOs, VIS, AUD, SSp and SSs. Scale bar: **(A)** 1 mm, **(B)** 100 μm. Abbreviations: olfactory areas (OLF), pallidum (PAL), striatum (STR), hypothalamus (HY), cortical subplate (CTXsp), hippocampal formation (HPF), thalamus (TH), midbrain (MB), pons (P), medulla (MY), cerebellum (CB), primary motor area (MOp), secondary motor area (MOs), visual area (VIS), auditory area (AUD), primary somatosensory area (SSp), and supplemental somatosensory area (SSs).

Generating a comprehensive catalog of CRH neurons requires detailed anatomical orientation. Co-located PI-stained cytoarchitecture acquired using BPS facilitated the precise segmentation of brain regions through co-registering with the template of Allen CCFv3. **Figure 4A** shows the registered region contours of three coronal planes in the same dataset. The outer profiles of the coronal planes showed good matching with the contours of Allen atlas (indicated by blue lines in **Figure 4A**). Distinct anatomical regions, such as the striatum, hippocampal formation and cerebellum, were identified. **Figure 4B** shows a dual-channel image of the partial enlarged view of **Figure 4A**. EYFP-labeled CRH neurons sparsely distributed in the cortex without distinct density differences, while PI-stained cytoarchitecture showed a typical laminated distribution, indicating different layers in the cortex (**Figure 4B**). Furthermore, we quantified cell densities with detailed anatomical subdivisions in the cortex in all datasets (**Figure 4C**). The results indicated that different sub-regions in the isocortex had a similar laminated distribution of CRH neurons. Layer 1 in the isocortex had the lowest cell density. The most abundant labeling of CRH was detected in Layer 2/3. Layer 4 also had relatively high expression, behind Layer 2/3. The cell densities of Layers 5 and 6 were close to or slightly higher than Layer 1. These results demonstrated the precise analysis of CRH expression with detailed anatomical annotation.

### Quantifying the Brain-Wide Distribution of CRH Neurons

**FIGURE 5:**
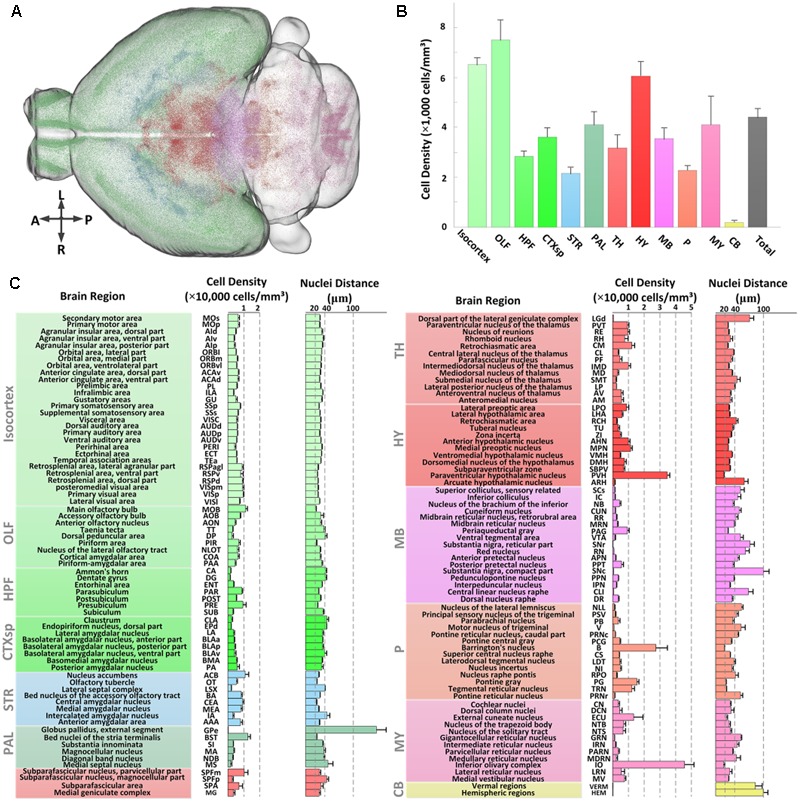
Quantitative statistics of the distribution of EYFP-labeled CRH neurons in the *CRH-IRES-Cre;Ai3* mouse brain. **(A)** Recognized positions of EYFP-labeled neurons in a *CRH-IRES-Cre;Ai3* mouse brain. Different color points represent the soma centers of CRH neurons in different regions. **(B)** Cell densities of EYFP-labeled CRH neurons in 12 brain regions. Color definitions are the same as those shown in **(A)**. **(C)** Cell density and nuclei distance of EYFP-labeled CRH neurons in the sub-regions of the above brain regions.

We first assessed the global distribution of CRH neurons in the brain (**Figure 5A**). The total number and average cell density of CRH neurons in the *CRH-IRES-Cre;Ai3* mouse brain were 760,865 ± 91,717 cells and 4,399 ± 358 cells/mm^3^ (*n* = 4), respectively. CRH neurons showed a brain-wide expression with significant regional differences (**Figure 5B**). Numerically, olfactory areas, isocortex, and hypothalamus had the three highest densities of CRH neurons, other areas had lower densities than the overall average. The density of CRH neurons in the pallidum, medulla, cortical subplate and midbrain was similar to that of entire brain. The thalamus, hippocampal formation had a lower density of CRH neurons than the whole brain. The expression of CRH in the pons and striatum was nearly half of the overall average. Specifically, only a small amount of CRH neurons were expressed in the cerebellum and the neuron density in the cerebellum was significantly lower than that in other brain areas. We further quantitatively analyzed the CRH distribution in 140 anatomical subdivisions (**Figure 5C**). The results showed that the PVH, B, and IO had the densest distribution of CRH expression, consistent with previous studies (Alon et al., 2009; Kono et al., 2016) and **Figures 2C,D**. The cell densities in these regions were higher than 20,000 cells/mm^3^. A total of nine sub-regions had neuron densities ranging from 10,000 to 20,000 cells/mm^3^. Among these sub-regions, the dense distribution of BST and nucleus accumbens was consistent with previous studies (Alon et al., 2009; Kono et al., 2016), while the MOB, subparafascicular nucleus, magnocellular part, central medial nucleus of the thalamus, MPN, PG, TRN, and ECU have received less attention. The densities in other sub-regions (128 sub-regions) were less than 10,000 cells/mm^3^. CRH neurons were locally and densely expressed in subareas in CEA and PIR (**Figures 2C–E**), with cell densities of only 9,250 ± 838 cells/mm^3^ and 6,891 ± 913 cells/mm^3^, respectively, reflecting the large full volume of the two regions. All the distribution features are relative consistent with that of *CRH-IRES-Cre;Ai14* mouse (The datasets are available in Allen Mouse Brain Atlas^1^). The inter-nuclear distances of CRH neurons were relatively negatively correlated with the cell density in most brain regions. The three regions had the densest distribution of CRH neurons also had the nearest inter-nuclear distances of the neurons, that is, under 20 μm. The nuclei distances in most regions (103 sub-regions) were between 20 and 40 μm. Among them, nuclei distances in MOB, CEA and PG were also under a relative low level (<22 μm). The distance of other regions are higher than 40 μm. Analysis of the distribution diversity of various regions will enhance the current understanding of CRH neurons and inform the functional study of CRH.

### Extracting the Morphological Characteristics of CRH Neuronal Somas

**FIGURE 6:**
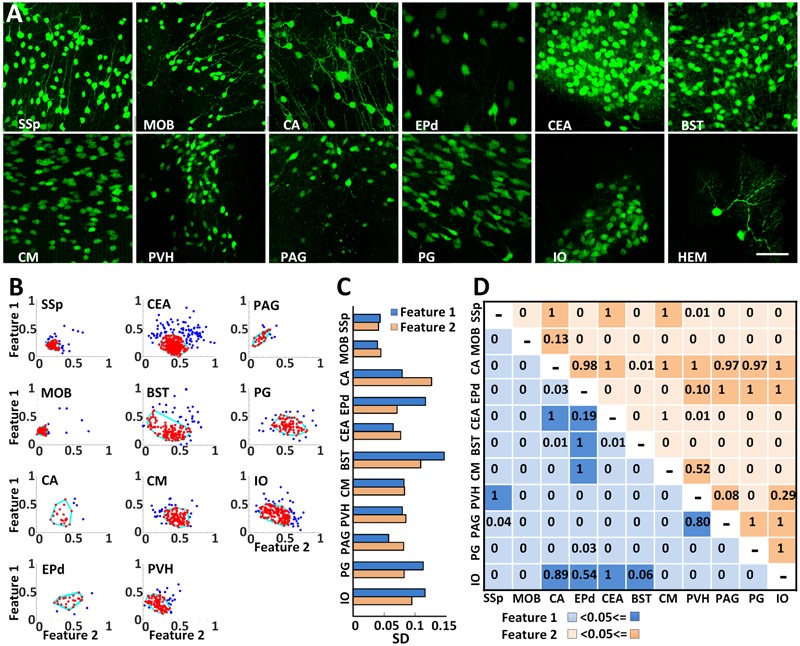
Analyzing soma morphology of EYFP-labeled CRH neurons in eleven discrete brain areas. **(A)** EYFP-labeled CRH neurons in 12 discrete brain areas. Each data cube was randomly selected at the size of 200 μm × 200 μm × 200 μm. Scale bar: 50 μm. **(B)** Extracting the morphological features of CRH neuronal somas using PCA transformation. Two normalized main features were acquired after PCA on the six morphological parameters. The cyan lines surrounding the most concentrated neurons with similar soma morphology account for 80% of the total neurons in each region. **(C)** SDs of both main features in the main regions except CB. **(D)** An ANOVA was conducted to analyze both of the main features. The light and dark colors represent *P* < 0.05 and *P* ≥ 0.05, respectively. Primary somatosensory area (SSp), MOB, Ammon’s horn (CA), endopiriform nucleus, dorsal part (EPd), CEA, bed nuclei of the stria terminalis (BST), central medial nucleus of the thalamus (CM), PVH, periaqueductal gray (PAG), PG, IO, and hemispheric regions (HEM).

High resolution BPS facilitated the analysis of the morphological similarities or differences of CRH neuronal somas in all twelve regions (**Figure 6A**). The soma sizes were relatively smaller in the primary somatosensory area, MOB, PVH, and periaqueductal gray, but larger in the other 8 regions. To characterize the soma morphology, we extracted the two main features using PCA from six original features of longest, shortest and average radii, surface area, cell volume and longest to shortest radii ratio (**Figure 6B**). As shown in **Figure 6C**, the SDs of both main features were less than 0.1 in the MOB, primary somatosensory area, CEA, central medial nucleus of the thalamus, PVH and periaqueductal gray, indicating that CRH neurons from the same region in these areas had a similar soma shape. One or both SDs of two main features in other regions were larger than 0.1. There might be subtypes in these regions, and further detailed analyses are needed. We also compared the soma morphology between different regions (**Figure 6D**). For the pairwise comparisons of the Ammon’s horn and BST, Ammon’s horn and IO, endopiriform nucleus, dorsal part and IO, and the periaqueductal gray and PVH, there was no significant difference in the two main features. The other pairs presented differently in at least one feature, indicating a shape diversity of CRH neuronal somas among different regions. This detailed morphological analysis facilitates the classification study of CRH neuron sub-types.

## Discussion

As an important neuropeptide, CRH is expressed throughout the whole brain. However, to date, the exact number, distribution density and morphological features of CRH neurons among various regions remains unknown. Technical advances in high-resolution whole-brain optical imaging and stereoscopic cell-counting algorithms have provided a great opportunity to reveal this neuroanatomical information. Here, we employed BPS and NeuroGPS to systematically study the CRH distribution at subcellular resolution with co-located cytoarchitecture in *CRH-IRES-Cre;Ai3* mouse brains. We acquired 4 datasets of EYFP-labeled CRH neurons and PI-counterstained anatomical references in *CRH-IRES-Cre;Ai3* mouse brains. We quantified and compared the distribution features of CRH expression and morphological features of CRH somas of different regions in the whole brain. These results will enhance the current understanding of how CRH neurons participate in specific functions.

The regional diversity of CRH expression in the present study provided an important anatomical reference for CRH-involved specific functions. Taking advantage of simultaneously acquired cytoarchitecture in the same brain, BPS guaranteed precise analysis of CRH neurons. As one of the most well-studied brain regions for CRH function, PVH is implicated in initiating the HPA axis neuroendocrine stress response (Dallman et al., 1992; Aguilera, 1994; Vaudry et al., 2013; Evanson and Herman, 2015). We observed dense distribution in the PVH. We also detected dense distribution of CRH in some specific regions, such as CEA, BST, and PIR, consistent with previous studies (Alon et al., 2009; Kono et al., 2016). The CRH neurons in above regions are implicated in the exposure to stress, fear or rewarding stimuli (Erb and Stewart, 1999; Zorrilla et al., 2014; Kono et al., 2016). In addition, systematic brain-wide screening revealed relatively high densities of CRH neurons in some regions that had not previously been explored, such as MOB, MPN, PG, TRN, ECU, and midline thalamus, indicating that more attention can be paid to these areas for the functional study of CRH neurons.

Furthermore, three-dimensional high-resolution imaging enabled the observation and reconstruction of the complete soma morphology of CRH neurons. The somas of CRH neurons exhibited a different size across brain regions. Specifically, the somas of CRH neurons in isocortex were relatively small and circular in shape compared to other areas. These neurons are mainly GABAergic cells and constitute a considerable fraction of the interneurons (Kubota et al., 2011; Taniguchi et al., 2011). In hippocampal formation, the CRH neuronal somas presented in a relative large polygonal shape (as shown in **Figures 2D, 6A**). Most neurons in this region are also GABAergic interneurons (Chen et al., 2004; Kono et al., 2016). The cell bodies were relatively large in CEA and IO and the CRH neurons may be GABAergic neurons and glutamate neurons, respectively (Kono et al., 2016). The soma reconstruction results quantitatively revealed the regional diversity of CRH neurons in both size and shape. In particular, the volumes of the CRH neurons in the PG, IO, CEA, and central medial nucleus of the thalamus were more than three times the volumes of the neurons in the MOB and periaqueductal gray. The soma shapes in the endopiriform nucleus, dorsal part, periaqueductal gray, PG, and IO were near ellipsoid, while those in the primary somatosensory area and MOB were close to circular. The PCA of the morphological features of CRH neuronal somas further demonstrated these diversities. These systematic visualization and comparison analyses have enabled the extraction of the detailed morphological characteristics of neurons. This technique potentially combined with electrophysiological recording and molecular phenotyping will offer a comprehensive indicator for the subtype classification of neurons.

We demonstrated a novel platform for visualizing, orienting and counting cells in three-dimension within the whole brain to quantify the brain-wide distribution of type-specific neurons. Compared with traditional two-dimensional imaging and counting, our method avoided the potential repeat counting of cells in adjacent slices or missed counting of axial-overlapped cells. To our knowledge, this study is the first to examine the exact number of CRH neurons in the *CRH-IRES-Cre;Ai3* mouse brain. It shows the unprecedented ability of automatic whole-brain optical imaging and stereoscopic cell counting and reconstructing to accurately and quantitatively detect and analyze the distribution and morphological features of specific neurons in the whole brain. We propose that this platform will become a routine tool for studying other type-specific neurons to enhance the current understanding of cell type.

## Author Contributions

JP, AL, HG, and JY designed the study. XL, BL, and JY performed tissue preparation and whole-brain data acquisition. XP, HN, and JP performed image processing and visualization. QL served as project advisor and participated in the planning and organizing of the project. JP, AL, and JY wrote the paper.

## Conflict of Interest Statement

The authors declare that the research was conducted in the absence of any commercial or financial relationships that could be construed as a potential conflict of interest.

## Footnotes

1 http://connectivity.brain-map.org
